# Dissecting *TET2* Regulatory Networks in Blood Differentiation and Cancer

**DOI:** 10.3390/cancers14030830

**Published:** 2022-02-06

**Authors:** Aleksey Lazarenkov, José Luis Sardina

**Affiliations:** Epigenetic Control of Haematopoiesis Group, Josep Carreras Leukaemia Research Institute, 08916 Badalona, Spain; alazarenkov@carrerasresearch.org

**Keywords:** DNA methylation, *TET2*, chromatin, transcription factors, gene regulation, blood malignancies

## Abstract

**Simple Summary:**

Bone marrow disorders such as leukemia and myelodysplastic syndromes are characterized by abnormal healthy blood cells production and function. Uncontrolled growth and impaired differentiation of white blood cells hinder the correct development of healthy cells in the bone marrow. One of the most frequent alterations that appear to initiate this deregulation and persist in leukemia patients are mutations in epigenetic regulators such as *TET2*. This review summarizes the latest molecular findings regarding *TET2* functions in hematopoietic cells and their potential implications in blood cancer origin and evolution. Our goal was to encompass and interlink up-to-date discoveries of the convoluted *TET2* functional network to provide a more precise overview of the leukemic burden of this protein.

**Abstract:**

Cytosine methylation (5mC) of CpG is the major epigenetic modification of mammalian DNA, playing essential roles during development and cancer. Although DNA methylation is generally associated with transcriptional repression, its role in gene regulation during cell fate decisions remains poorly understood. DNA demethylation can be either passive or active when initiated by TET dioxygenases. During active demethylation, transcription factors (TFs) recruit TET enzymes (TET1, 2, and 3) to specific gene regulatory regions to first catalyze the oxidation of 5mC to 5-hydroxymethylcytosine (5hmC) and subsequently to higher oxidized cytosine derivatives. Only *TET2* is frequently mutated in the hematopoietic system from the three TET family members. These mutations initially lead to the hematopoietic stem cells (HSCs) compartment expansion, eventually evolving to give rise to a wide range of blood malignancies. This review focuses on recent advances in characterizing the main *TET2*-mediated molecular mechanisms that activate aberrant transcriptional programs in blood cancer onset and development. In addition, we discuss some of the key outstanding questions in the field.

## 1. Introduction

CpG methylation is the most common DNA modification found in the mammalian genome [1], playing an essential role in development and cancer [2,3]. DNA demethylation can be either passive, by dilution of DNA methylation after each cell division, or active when initiated by Tet dioxygenases [4]. During active demethylation, the Ten-eleven-translocation (TET) family of enzymes first catalyze the iterative oxidation of 5-methylcytosine (5mC) to 5-hydroxymethylcytosine (5hmC) and subsequently to higher oxidized derivatives, 5-formylcytosine (5fC) and 5-carboxycytosine (5CaC) [5,6,7]. These higher oxidized forms of cytosine, in turn, can either be lost during replication or enzymatically removed, restoring unmodified cytosine and alleviating transcriptional repression typically associated with 5mC residues [4]. However, 5hmC and higher oxidized derivatives should be considered not only as mere transient states in the DNA demethylation path but as bona fide epigenetic marks as illustrated by their complex network of specialized readers [8].

In the context of the hematopoietic system, TET family members have been involved in naturally occurring and experimentally induced cell fate decisions [9,10,11,12,13,14]. Among the TETs, *TET2* is the most broadly expressed and frequently mutated gene in blood malignancies [15,16,17,18,19,20]. *TET2* mutational profiles show inactivating mutations occurring along the whole gene coding region and are not only restricted at the 3′ region where the catalytic domain is located [15,17,21]. Therefore, *TET2* epigenetic regulation during blood differentiation might be mediated not only by its catalytic activity but also by its association with critical partners [22,23].

Here we review the current understanding of *TET2* functions in normal and malignant hematopoiesis, providing an extensive overview of the intricate molecular mechanisms controlling gene expression, protein stability and function, and enzyme’s genome recruitment.

## 2. Mechanisms of *TET2* Protein/Enzymatic Regulation

Several mechanisms regulating *TET2* expression and activity have been elucidated in the last decade. Here we summarize main control systems, encompassing basal post/transcriptional regulation, direct protein modulation through post-translation modifications (PTMs), and enzymatic substrate availability.

### 2.1. Transcriptional Regulation

Some transcriptional factors have been defined as direct regulators of TETs’ gene expression. In mouse embryonic stem cells (ESCs), *Tet1* and *Tet2* are positively regulated by the pluripotency TF Oct4 that binds to conserved non-coding sequences in both genes [24]. Accordingly, upon ESC differentiation, *Tet1* and *Tet2* levels decrease due to Oct4 depletion [24].

A similar regulatory mechanism was described for the CXXC-DNA binding domain protein Rinf (CXXC5), whose depletion leads to decreased *Tet1* and *Tet2* expression [25]. During myeloid cell fate commitment, *Tet2* expression is boosted by the action of the myeloid transcription factor CEBPα, which binds to *Tet2* enhancer regions [26]. CEBPα might exert its transcriptional control by recruiting *Tet2* protein itself to *Tet2* gene’s distal regulatory regions leading to their demethylation and activation [10]. Of note, a mutant form of CEBPα (Brm2), recapitulating naturally occurring mutations in AML patients [27], failed to demethylate the *Tet2* enhancers [10]. In addition, histone deacetylase 4 (HDAC4) protein has been recently described as a positive regulator of *TET2* expression in the context of MDS and AML [28]. Finally, in regulatory T cells (Treg), *Tet1* and *Tet2* are regulated in response to hydrogen sulfide (H2S) through the action of the sulfhydrating nuclear transcription factor Y subunit beta (NFYB) [29].

### 2.2. Post-Transcriptional Regulation

miRNAs regulatory networks targeting *TET2* mRNAs have been proposed as the primary post-transcriptionally regulatory mechanism during blood differentiation and in myeloid malignancies. High-throughput screens identified a large subset of *TET2* 3′UTR targeting miRNAs with different efficiencies. Induced expression of those led to an array of leukemic traits such as myeloid lineage bias, phenocopying to an extent, a direct *TET2* loss [30]. In a related study, miR-22 (also targeting *TET2* mRNA) was detected overexpressed in MDS patients. Mechanistically, miR-22 overexpression leads to reduced genome-wide levels of 5hmC, increased self-renewal, and myeloid skewing [31]. Also, in MDS, miR-9 and miR-34a indirectly control *TET2* by post-transcriptionally regulating SIRT1 levels, which affect the *TET2* protein function at a post-translational level (See Section 2.3) [32].

### 2.3. Post-Translational Regulation

Although *TET2* protein levels are mainly regulated via transcriptional mechanisms, *TET2* post-translational modifications might be involved in rapidly fine-tuning protein levels in response to external cues.

During ESC differentiation, the CXXC-DNA binding domain protein IDAX (CXXC4) recruits *TET2* to DNA, activating caspases that cleave the *TET2*-IDAX complex leading to *TET2* protein depletion [33]. Similarly, TET proteins have been described as direct substrates of the calpain family of proteases [34]. Calpain1 regulates the degradation of *Tet1* and *Tet2* in mouse pluripotent ESCs and calpain2 of Tet3 during ESCs neural differentiation. These negative regulatory mechanisms ensure correct global 5hmC level maintenance and expression of lineage-specific genes in ESCs [34].

**Figure 1:**
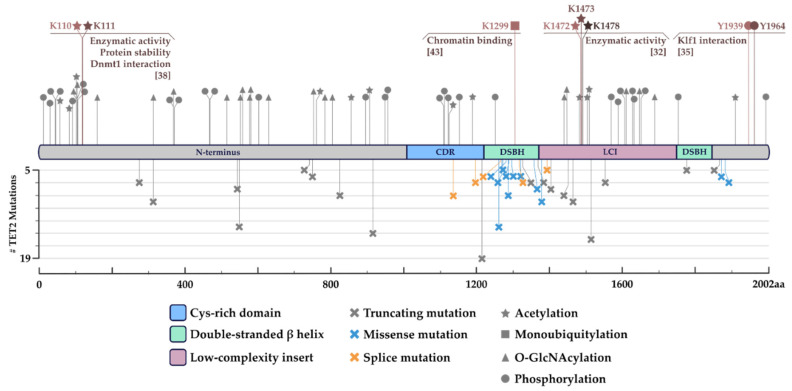
Schematic representation of *TET2* post-translational modifications (PTMs) and most frequently found *TET2* mutations. In the upper half, compiled PTM data based on mass spectrometry (MS) and in silico predictions [35,36,38,43,44]. In grey, proposed modifications with an undefined function. In color, fully characterized PTMs and their impact on *TET2* activity. In the bottom half, a compilation of *TET2*′s most frequent mutations and their type (truncating, missense, or splice mutations). Filtering was done from combined 12,845 samples from 35 studies where the mutation was found in >5 different patients. Data from ‘Myeloid’ dataset from cBioPortal (https://www.cbioportal.org/, accessed on 19 January 2022) [45,46].

In addition, *TET2* protein can be largely post-translationally modified (PTM) by (de)phosphorylation, (de)acetylation, O-GlcNAcylation, or ubiquitylation, among others [32,35,36,37,38] (Figure 1). The specific output driven by particular PTMs on *TET2* activity is cell type and amino acid residue-specific. For instance, cytokine receptor-associated JAK2 (in response to FLT3 or EPO/SCF signaling in blood progenitor cells) phosphorylates *TET2* at tyrosine residues 1939 and 1964, leading to enhanced enzymatic activity [35] (Figure 1). However, *TET2* tyrosine residues 1939 and 1964 are not the only phosphorylatable residues since the whole N-terminus region of the protein (constituting a large disordered domain) is usually highly phosphorylated in ESCs [36] (Figure 1). Interestingly, the O-linked N-acetylglucosaminyltransferase (OGT), a strong TET interactor [36,39,40,41,42], adds O-GlcNAcylation groups to serine and threonine residues of TET2, thereby reducing the number of available phosphorylation sites and their site occupancy [36]. Once again, the described phosphorylation vs. O-GlcNAcylation mechanism highlights the fine-tuning TET proteins might undergo for correct localization and activity according to external signals [36].

TET2 activity can also be regulated through protein (de)acetylases. This is the case of the NAD-dependent deacetylase sirtuin-1 (SIRT1) that removes acetylation at *TET2* specific lysine residues K1472, K1473, and K1478, increasing protein’s enzymatic activity [32]. Consequently, reduced SIRT1 activity in human HSPCs leads to the onset of an MDS-like disease recapitulating the phenotype observed in *TET2*-mutated MDS patients [32]. Whereas *TET2* global deacetylation mediated by histone deacetylases, 1 and 2 (HDAC1 and 2) leads to reduced enzymatic activity triggering the emergence of abnormal DNA methylation profiles typically associated with cancer [38]. Zhang and co-workers also studied the effects on *TET2* stability/activity mediated by histone acetyltransferase p300 (enzymatic counterpart of the HDAC1/2 enzymes). p300 was shown to acetylate the *TET2* N-terminus region leading to increased protein activity, stability, and partnering with other proteins such as DNMT1 [38]. TET2/DNMT1 complex might prevent abnormal promoter methylation typically observed upon exposure to oxidative stress [38].

Finally, ubiquitylation of *TET2* has been described to regulate its chromatin association [43]. CRL4 (VprBP) E3 ligase, a member of the ubiquitin ligase complex, has been shown to interact with the cysteine-rich domain of *TET2* and promote K1299 monoubiquitylation, enhancing its chromatin association [43]. On the contrary, USP15-dependent K1299 deubiquitylation leads to decreased *TET2* activity [44].

### 2.4. Enzymatic Regulation

Regarding catalytic activity, TET enzymes are Fe2+/α-KG-dependent dioxygenases. Metabolite and cofactor availability constitute another relevant layer of protein regulation potentially influencing hematopoiesis and leukemic development. Interestingly, experimentally-induced ascorbate (VitC) depletion leads to increased HSC function and compartment expansion, resembling the aberrant self-renewal phenotype typically observed upon *Tet2* depletion in HSCs [47,48,49]. In addition, Cimmino and co-workers elegantly demonstrated the potential of VitC treatment to rescue an aberrant self-renewal phenotype initiated upon *Tet2* in vivo depletion [50]. On the contrary, 2-Hydroxyglutarate (2-HG), an oncometabolite produced in *IDH1/2* mutated patient cells, competitively inhibits *TET2* catalytic activity [50,51], resulting in genome-wide DNA hypermethylation and impaired myeloid differentiation [52]. Similarly, mutations in other metabolic players such as *fumarate hydratase (FH)* and *succinate dehydrogenase (SDH)* lead to fumarate and succinate accumulation that inhibit Tet enzymatic activity even with stable α-KG levels [53]. The interplay between these metabolic intermediaries and *TET2* might also be directly relevant in clinics as mutations in iron and 2-oxoglutarate-binding sites have been reported in AML patients [15,54]. Finally, although essential for TET catalytic activity, oxygen has been described as a minor direct regulator of TET function in physiological settings [55]. However, low oxygen levels might indirectly regulate *TET2* expression and activity in leukemic cells through a mechanism involving the activation of the hypoxia-inducible factor 1α (HIF1α) [56].

## 3. Partner-Instructed *Tet2* Genomic Recruitment during Development and Cancer

Regulation of TET activity by controlling enzymes’ genomic distribution allows surgically modifying DNA methylation at particular genomic loci and only in specific cell types. Since *TET2* lacks the low-affinity (CXXC) DNA binding domain, which is present in *TET1* and TET3 [33], the enzyme must always be recruited to specific genomic locations through a ‘partner’ protein such as transcription factors.

**Figure 2:**
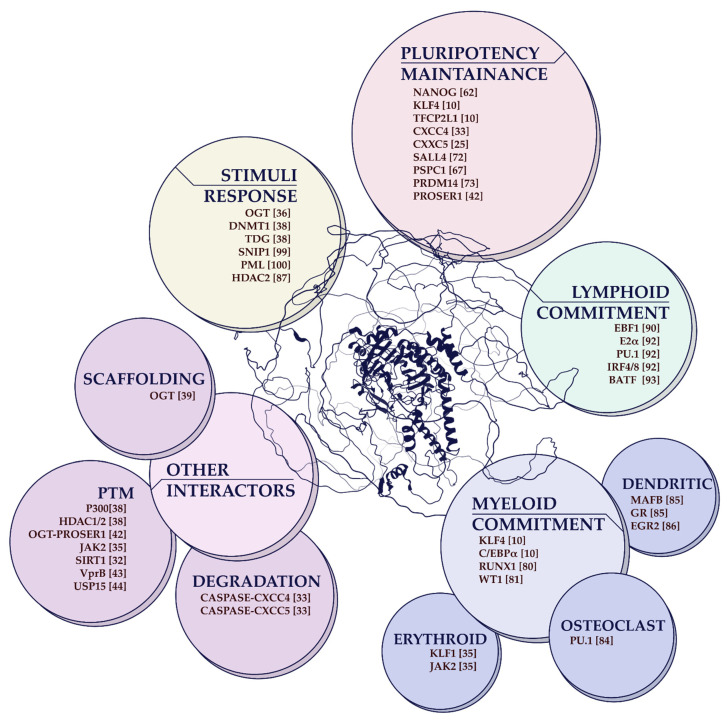
Graphical overview of *TET2* protein partners/interactors. In the center, the human *TET2* AlphaFold Database predicted protein structure (last updated on 1 July 2021 with AlphaFold v2.0) [57,58]. Encompassing the *TET2* protein structure, color-coded bubbles cluster *TET2* interactors based on previously described cell fate implications and physiological responses when cooperating with *TET2*. GR: glucocorticoid receptor.

Here we present an elaborated list of potential *Tet2* interactors/recruiters playing relevant roles in different biological settings (Figure 2).

### 3.1. During Embryonic Stem Cell Fate Decisions

Tet2 is considered an important pluripotency regulator playing critical roles during experimentally-induced pluripotency establishment [10,59,60,61,62,63,64] and in pluripotency maintenance [65,66,67]. In addition, Tet enzymes are essential for proper pluripotency exit during embryo development [68,69] and in ES cell differentiation [70,71].

Molecular mechanisms underlying the *Tet2* pluripotency-related functions have recently been partially uncovered by systematically identifying and characterizing critical *Tet2* interactors in ESCs. The naïve pluripotency TF Nanog was the first pluripotency-related protein identified interacting with *Tet2* and *Tet1* [62]. Thus, explaining *Tet2* and *Tet1* bound at regulatory regions of pluripotency genes in ESCs [62,66]. However, *Tet2* is also functionally associated with other pluripotency-related TF at these regions, including Sall4 [72] and Prdm14 [73]. Sall4 acts in concert with *Tet1* to firstly oxidize 5mC CpG residues into 5hmC and later with *Tet2* to further oxidize them into 5fC and 5caC residues. However, no direct physical interaction between Sall4 and *Tet2* was observed in ESCs [72]. Thus, suggesting the involvement of additional factors in this functional interplay. On the contrary, Prdm14, through direct association with Tet2, drives active demethylation at pluripotency and germline-associated genes such as *Tcl1, Tcfap2c,* and *Spo11, Sycp3*, respectively [73].

Additionally, *Tet2* has been described to interact with a handful of non-canonical pluripotency TFs equally playing essential roles in regulating its functions in ESCs. The latter includes the CXXC-DNA binding domain proteins Idax (CXXC4) and Rinf (CXXC5), potentially influencing *Tet2* functions in differentiation [33] and pluripotency [25], respectively. Rinf co-occupies with *Tet2* and Nanog distal regulatory regions of relevant pluripotency genes (such as Oct3/4, Sox2, and Nanog itself), positively regulating their transcription. The RNA-binding protein PSPC1 associates with *Tet2* and targets the enzyme to the MERVL endogenous retroviral elements in ESCs [67]. Of note, these retroviral elements have been described to regulate the expression of 2C-genes in ESCs [74,75]. Once recruited to MERVL elements, *Tet2* contributes to their transcriptional and epitranscriptomic regulation by recruiting HDACs to chromatin and oxidizing MERVL transcripts, respectively [67].

As mentioned above, *Tet2* is also required during experimentally induced pluripotency establishment. To uncover factors associated with *Tet2* in this biological setting, we analyzed DNA (hydroxy)methylation dynamics during rapid and highly efficient iPSC reprogramming systems [59,60,76]. Here, *Tet2* is recruited by the Klf4 and Tfcp2lƒ1 transcription factors to drive active enhancer demethylation of chromatin and pluripotency-related genes to reprogram cell fate [10].

Of note, a prominent study has recently identified common molecular mechanisms controlling DNA methylation during embryo development and in the leukemic transformation [77]. Thus, highlighting the potential health implications of studying Tet-mediated DNA demethylation in embryonic stem cells.

### 3.2. During Blood Cell Fate Decisions

Tet2 role in hematopoiesis and its influence on the DNA methylome has also been widely explored in the context of physiological and pathological conditions. Several studies have described how *Tet2* protein deficiency can lead to aberrant cell fate decisions by extensively altering the DNA methylome during hematopoiesis. Here we recapitulate the role of *Tet2* recruitment to specific genomic regions in different hematopoietic developmental pathways: 

#### 3.2.1. During Myeloid Cell Fate Decisions

Tet2-mediated epigenetic gene regulation is crucial during myeloid cell development. C/EBPα is an essential factor in the differentiation process from HSPCs to GMPs [78]. C/EBPα alone, through its pioneer activity, or in concert with PU.1, binds regulatory regions of myeloid genes to establish the myeloid cell fate [79]. To this end, C/EBPα directly activates *Tet2* expression [26] and, through direct interaction with the enzyme, targets it to regulatory regions of myeloid genes such as *Klf4* or *Chd7*, driving their active DNA demethylation and subsequent enhancer activation [10]. RUNX1, another key hematopoietic transcription factor, has also been described to interact with *TET2* [80]. Thus, it potentially leads to the DNA demethylation observed at RUNX1 binding sites (including promoters of *PTPN22*, *RUNX1*, and *RUNX3*, among others) during hematopoietic differentiation [80]. Wilm’s tumor (*WT1*) gene encodes a sequence-specific transcription factor found mutated in a mutually exclusive manner with *TET2* in AML patients [20]. Interestingly, WT1 directly associates with *TET2* [81] to regulate WT1-target genes (including Wnt and MAPK signaling-related genes such as *BTRC*, *DACT1*, and *TBL1X*), preventing AML onset [81,82].

However, *TET2* activity is not only relevant during early myeloid commitment but also in myeloid terminal differentiation [83]. In this regard, during monocyte-to-osteoclast differentiation, the master myeloid TF PU.1 recruits *TET2* to promoters of key osteoclast-genes (including *ACP5*, *CTSK*, and *TM7SF4*), leading to their demethylation and cell fate transition [84]. Tolerogenic dendritic cells (tolDCs) are also terminally differentiated myeloid cells with potent immunosuppressive properties. Mechanistically, the tolerogenic phenotype is acquired through a synergistic interplay between the glucocorticoid receptor (GR) and the specific myeloid transcription factor MAFB. Both TFs target *TET2* at genomic loci exclusively demethylated in tolDCs [85]. In addition, EGR2, an essential transcription factor during IL4/GM-CSF-driven monocyte (MO) to monocyte-derived DCs (moDCs) differentiation, has also been described to interact with *TET2* and initiate DNA demethylation at both EGR2 stable and transient binding sites [86]. However, *TET2* genomic recruitment is not always associated with positive regulation of gene expression in dendritic cells. For instance, Zhang and co-workers identified that IκBζ-dependent *Tet2* targeting the *Il6* locus leads to Hdac2 recruitment. Thus, finally leading to *Il6* gene repression and inflammation resolution [87].

#### 3.2.2. Erythroid Lineage

HSPC commitment towards erythroid lineage also correlates with 5hmC accumulation and increased expression of erythroid-specific genes such as *EPOR*, *GATA1*, and *HBB* [88]. 5hmC accumulation might be mediated by Klf1-dependent *Tet2* recruitment at erythroid-specific genes. Of note, both factors have been recently described to interact upon Jak2-driven *Tet2* phosphorylation [35]. 

#### 3.2.3. B-Cell Lineage

B cell differentiation is tightly regulated at the epigenetic level. B cell maturation is characterized by extensive reshaping of the cellular methylome [89]. *Tet2* might contribute to the process by interacting with B-cell master regulators such as EBF1 [90], IRF4/8, E2A, and PU.1 or BATF and driving focal demethylation at regulatory regions of key B-cell loci (including *IgK* or *Aicda)* [91,92,93].

#### 3.2.4. T-Cell Lineage

5hmC is accumulated at genes encoding key regulators of T cell identity, development, and differentiation [94]. However, what factors recruit Tet enzymes to the T-cell key regulatory regions is poorly known. In regulatory T cells (Tregs), *Tet1* and *Tet2* are upregulated in response to the sulfhydrating nuclear transcription factor Y subunit beta (NFYB) [29]. *Tet1* and *Tet2* are targeted by the activated forms of Smad3 and Stat5 to the Foxp3 promoter favoring its hypomethylation and stable gene expression [29].

### 3.3. In Response to External Stimuli

Tet2 has also been implicated in genome stability. Upon exposure to oxidative stress (OS), protein complexes containing DNMTs and *TET2* are recruited to the specific genomic regions to repair the damaged DNA properly. *TET2* depletion leads to reduced 5hmC levels at the damaged regions and impaired repair [95,96,97,98]. As a result of these exciting findings, several epigenetic regulatory mechanisms that tie *Tet2* to protection against abnormal DNA methylation during stress have been proposed. For instance, Zhang and co-workers showed that upon OS, *TET2* interacts with the thymidine glycosylase (TDG), an enzyme involved in the DNA active demethylation pathway. Then, both proteins are recruited to chromatin in a DNMT1 dependent manner, a process that is enhanced when *TET2* is acetylated [38]. Therefore, the latter mechanism is suggested to protect against the acquisition of abnormal DNA methylation at typically unmethylated gene regulatory regions. 

Another *Tet2* interactor in the context of DNA damage response is the SMAD nuclear interacting protein 1 (SNIP1) that regulates the expression of relevant c-MYC target genes involved in apoptosis. Therefore, reduced SNIP1 levels lead to diminished 5hmC levels and gene expression at c-MYC target genes [99].

Finally, chemotherapeutic drugs have also been described to trigger hydroxymethylation changes in a mechanism mediated by the interaction between the promyelocytic leukemia protein (PML) and TET2, linking the protein to direct clinical applications [100].

## 4. *TET2* Loss of Function in Blood Malignancies

DNA methylation aberrations are considered hallmarks of cancer onset and progression [101,102,103]. *TET2* loss of function mutations are frequently found in patients suffering from myeloid malignancies such as acute myeloid leukemia, myeloproliferative neoplasms, or myelodysplastic syndromes [15,20,104] (Table 1). However, these mutations are also commonly observed in other blood malignancies, including B and T lymphomas [105,106,107,108,109], such as the angioimmunoblastic T-cell lymphoma (AITL), showing the highest incidence of *TET2* mutations (roughly 80%) among all blood cancer types (Table 1). The broad *TET2* mutational profile observed in the hematopoietic system has awakened great interest in the field to unravel the molecular mechanisms underlying *TET2* involvement in the onset of preleukemic and leukemic diseases.

### 4.1. Preleukemic Conditions

HSCs, like any other somatic cells, slowly accumulate mutations during the whole life of an individual [121]. *TET2* mutated HSC clones are frequently found among healthy aged individuals characterized by an increased risk of cancer onset and a higher propensity to develop cardiovascular diseases [18,122,123,124]. This condition is named clonal hematopoiesis of indeterminate potential (CHIP). Mechanistically, *TET2* loss of function mutations appear to enhance HSCs’ expansion capabilities and lead to a myeloid bias [48,52,125,126,127]. Additionally, *TET2* mutant cells show an enhanced response to pro-inflammatory cytokines such as IL-6 and TNF-α [128], potentially representing a competitive advantage in response to inflammatory environments. Moreover, inflammation-based preleukemic myeloproliferation has been linked to *TET2* activity [129,130], where *Il6* expression might be negatively regulated by Tet2-mediated Hdac2 recruitment to its promoter [87].

Regardless, HSCs with altered *TET2* activity acquire a competitive clonal advantage due to an altered DNA methylation landscape that finally allows aberrant gene expression. However, the precise order of events leading from CHIP to the development of myeloid malignancies is not yet fully uncovered, nor is the CHIP potential as a clinical predictor.

### 4.2. Leukemic Conditions

As previously mentioned, *TET2* loss of function mutations are frequently found in patients with myeloid malignancies, myeloproliferative disorders (MPN), or myelodysplastic syndromes (MDS) (Table 1). These conditions are typically considered as an early event in myeloid cancer development [15,131] and are characterized by presenting aberrant DNA methylation profiles due to incorrect *TET2* function [52,103]. This is the case, for instance, of chronic myelomonocytic leukemia (CMML), a mixed MDS/MPN neoplasm where *TET2* mutations are present in up to 60% of patients [16,132] (Table 1). In CMML cells, aberrant methylation was observed at promoters of genes involved in neoplastic transformation, WNT and PDGF signaling pathways; inflammation; and apoptosis [133]. Of note, similar gene sets were observed aberrantly methylated and expressed in *TET2*-mutated AML patients, including genes coding for tumor suppressors (*PDZD2*); transcription modulators (*ZNF667*, *ZNF582*, *PIAS2*, *CDK8*); nuclear import receptors (*TNPO3*, *IPO8*); and myeloid cytokines (*CSDA*) [103].

Global methylation analysis also revealed a shared hypermethylation signature in a subset of AML patients carrying *IDH1/2* mutations [52,134], a well-known *TET2* inhibitor and mutually exclusive mutated gene (See Section 2.4). The latter highlights the importance of *TET2* enzymatic deregulation.

Similarly, mutations in the IDH1/2-*TET2*-WT1 network, which collectively appear in 30-50% of AML cases [20,81,131], also present an apparent hypermethylated phenotype as a consequence of deficient *TET2* activity [82,135]. Several ongoing clinical trials aim to restore *TET2* activity within the IDH1/2-*TET2*-WT1 mutated network by combining hypomethylating agents with ascorbate treatment [50,51,135,136,137] (ClinicalTrials.gov identifier: NCT03999723, NCT03682029).

In the lymphoid lineage, aberrant B cell differentiation and chronic lymphocytic leukemia (CLL) have been associated with DNA hypomethylation [138,139,140]. Hypomethylation at gene bodies and enhancer regions correlates with gene expression differences in CLL samples compared to normal B cells [89,139,141]. However, available data do not support a primary role of TET enzymes in CLL, but perhaps an accessory role in establishing leukemia-specific patterns of 3D chromatin conformation. The latter might be accomplished, for instance, by modulating CTCF binding to the chromatin, a DNA methylation-sensitive mechanism [142].

TET2 has also been studied in the context of lymphoproliferative diseases. Diffuse large B-cell lymphoma (DLBCL) patients have shown specific hypermethylation signatures on promoters of tumor suppressor genes involved in cell fate and cell cycle changes [109]. Germinal center analysis of B cells in *Tet2* deficient mice showed promoter hypermethylation and defective transcription factor binding at essential B-cell pathway genes. *Tet2* knockout mice partially phenocopied DLBCL patients, characterized by downregulation of antigen presentation genes/interferon pathway, lymphoma-like transcriptional profiles similar to CREBBP-mutant patients, and a failure at the germinal center exit. Therefore, this data suggests that *TET2* is relevant in B cell lymphoma development and how acquiring mutations in HSCs might influence B-cell maturation and cancer development [105,106].

## 5. Summary and Conclusions

The last decade of research has shed light on the complex biological functions of *TET2* in normal hematopoiesis and blood cancer development. However, as described in this manuscript, a holistic approach is required to deconvolute the intricated regulatory networks determining *TET2* function in a cell type-specific manner. Precisely, *TET2* activity is controlled by: (1) *TET2* transcriptional regulation (through Oct4, C/EBPα, Rinf or HDAC4); (2) post-transcriptional regulation (by miR-22, miR-9, or miR-34a); (3) post-translational regulation (by a whole array of PTMs (Figure 1); (4) enzymatic regulation (by VitC, α-KG/2-HG, FH, SDH or oxygen/HIF1α) and (5) genomic localization (through interactions with an extensive network of partners (Figure 2).

C/EBPα regulating *Tet2* gene expression by directly recruiting *Tet2* enzyme to demethylate its own enhancers during myeloid cell fate specification [10] perfectly illustrates the complexity of the *Tet2* regulatory networks. Another interesting example of multilayer regulation is the miR-9/miR-34a-SIRT1-TET2 network. Sun and co-workers identified miR-9/miR-34a overexpression within a subset of *TET2*-WT MDS patients displaying a *TET2* loss of function phenotype [32]. miR-9/miR-34a post-transcriptionally regulates SIRT1, which modulates *TET2* activity by mediating deacetylation at key lysine residues of the enzyme [32]. 

About *TET2* post-translational regulation, a comprehensive characterization of all potential PTMs described (Figure 1) is needed to fully determine their physiological relevance. Enhanced knowledge of TET2′s PTM-ome might be particularly relevant to fully elucidate the functions mediated by well-known *TET2* interactors such as SIRT1 or OGT. Factors that can modify *TET2* enzymatic capabilities and modulate its interaction with other proteins [32,37,38,39,40,42,43] (Figure 1).

Substrate availability is the primary mechanism of enzymatic regulation. Therefore, *TET2* activity is tightly regulated by the availability of α-KG and 2-HG produced by IDH1/2-WT or -mutated enzymes, respectively [50,51]. First studies conducted in AML patient cells harboring 2-HG-producing *IDH1/2* mutations uncovered an altered cellular epigenetic landscape and a myeloid bias characteristic of AML patients with *TET2* loss of function [52,90,134,143]. However, such myeloid bias has not been observed when analyzing the multilayer differentiation potential of *Idh2* mutated cells through a single-cell approach [144]. The apparent discrepancy observed in the phenotype might be explained by species-specific differences, variations in 2-HG levels produced; differences in 2-HG subtype (D-2-HG or L-2-HG) produced; or a side-effect of 2-HG-mediated inactivation of other α-KG-dependent dioxygenases such as the Jumonji-C domain histone demethylase family. 

The rapidly increasing list of partners/interactors identified highlights the idea that *TET2* has been promiscuously recruited to the genome by sequence-specific transcription factors (also by other epigenetic regulators including HDAC1/2 or DNMT1) to drive active DNA demethylation in a cell state-specific manner. *TET2* promiscuous behavior might be encoded in its nucleotide sequence where only part of the catalytic domain (coding for the cysteine-rich domain and the first double-stranded β-helix) is structured and ordered (Figure 1 and Figure 2 and in silico prediction). Whereas the N-terminus protein region is unstructured, therefore, constituting a large intrinsically disordered domain (IDR) that might favor most *TET2* described interactions through a mechanism of liquid-liquid phase separation (LLPS). Of note, IDR-mediated LLPS events have been recently described for the *TET2* recruiter KLF4 and its associated pluripotency transcription factor OCT4 [145,146]. 

To sum up, fine dissection of the molecular mechanisms underlying *TET2* activity is essential to fully uncover its contribution to clonal expansion and malignant transformation.

## Figures and Tables

**Table 1:** List of *TET2* mutation frequencies in hematologic malignancies and their prognostic value. Combined 12845 samples from 35 studies were analyzed according to cancer type and detailed classification based on WHO Classification of Hematologic Malignancies or The French–American–British (FAB) classification. Data from ‘Lymphoid’ and ‘Myeloid’ datasets from cBioPortal (https://www.cbioportal.org/, accessed on 19 January 2022) [45,46] and selected referenced studies [108,110,111,112]. AML = Acute myeloid leukemia, AMML = Acute Myelomonocytic Leukemia, AML-M5 = Acute Monoblastic/Monocytic Leukemia, CML = Chronic Myelogenous Leukemia, MPN = Myeloproliferative Neoplasms, MDS = Myelodysplastic Syndromes, CMML = Chronic Myelomonocytic Leukemia, CLL/SLL = Chronic Lymphocytic Leukemia/Small Lymphocytic Lymphoma, DLBCL = Diffuse Large B-Cell Lymphoma, AITL = Angioimmunoblastic T-cell lymphoma, NOS = Not Otherwise Specified, N/A = Not Available.

Cancer Type	Detailed Cancer Type	Frequency (%)	Prognosis
AML (399/4014) 10%	AML (unspecified)	9	Unfavorable [113]
	AML, NOS	17	Unfavorable [113]
	AML with Biallelic Mutations of CEBPA	24	Unfavorable [114]
	AML with inv (3) (q21.3q26.2) or t(3;3) (q21.3;q26.2); GATA2, MECOM	8	N/A
	AML with mutated NPM1	18	Unfavorable [19]
	AML with Myelodysplasia Related Changes	8	Unaffected [115]
	AML with Recurrent Genetic Abnormalities	5	N/A
	AML with t(8;21) (q22;q22.1); RUNX1-RUNX1T1	13	N/A
	AMML	8	N/A
	AML-M5	15	N/A
MDS/MPN (1023/2700) 38%	MDS (unspecified)	25	Favorable [116]
	MDS, Unclassifiable	13	Favorable [116]
	MDS with Excess Blasts (unspecified)	22	Favorable [116]
	MDS with excess blasts-1	21	Favorable [116]
	MDS with excess blasts-2	16	Favorable [116]
	MDS with isolated del(5q)	16	Unaffected [117]
	MDS with Multilineage Dysplasia	33	Favorable [116]
	MDS with Single Lineage Dysplasia	20	N/A
	MDS/MPN with Ring Sideroblasts and Thrombocytosis	28	N/A
	MPN	22	Unaffected [118]
	CMML	56	Favorable [118]
	CML	30	Unaffected [119]
	Essential thrombocythemia	9	Unaffected [54]
	Polycythemia Vera	28	Unaffected [54]
	Primary myelofibrosis	26	Unaffected [54]
	Histiocytic and Dendritic Cell Neoplasms	2	N/A
B/T-cell neoplasms (343/3712) 9%	Burkitt Lymphoma	4	N/A
	DLBCL, NOS	6	N/A
	DLBCL (unspecified)	11	Favorable [120]
	DLBCL, Germinal Center B-Cell Type	7	N/A
	DLBCL, Activated B-cell Type	1	N/A
	Follicular Lymphoma	4	N/A
	High-Grade B-Cell Lymphoma, NOS	5	N/A
	Mantle Cell Lymphoma	3	N/A
	Marginal Zone Lymphoma	4	N/A
	Mature B-Cell Neoplasms	16	N/A
	AITL	78	Unaffected [108]
	CLL/SLL	1	N/A
	Sezary Syndrome	12	N/A
Therapy-Related Neoplasms (12/115) 10%	Therapy-Related Myeloid Neoplasms (unspecified)	8	N/A
	Therapy-Related Myelodysplastic Syndrome	27	N/A
